# Evaluation of Different Shallow Groundwater Tables and Alfalfa Cultivars for Forage Yield and Nutritional Value in Coastal Saline Soil of North China

**DOI:** 10.3390/life12020217

**Published:** 2022-01-30

**Authors:** Shichao Wang, Kai Guo, Asif Ameen, Dong Fang, Xiaolin Li, Xiaojing Liu, Lipu Han

**Affiliations:** 1 Key Laboratory of Agricultural Water Resources, Hebei Key Laboratory of Soil Ecology, CAS Engineering Laboratory for Efficient Utilization of Saline Resources, Center for Agricultural Resources Research, Institute of Genetics and Developmental Biology, Chinese Academy of Sciences, Shijiazhuang 050022, China; scwang@sjziam.ac.cn (S.W.); guokai@sjziam.ac.cn (K.G.); fangdong19@mails.ucas.ac.cn (D.F.); xlli@ms.sjziam.ac.cn (X.L.); xjliu@sjziam.ac.cn (X.L.); 2 Rice Programme, Pakistan Agricultural Research Council, Kala Shah Kaku, Lahore 39020, Pakistan; asifameen2007@gmail.com; 3 University of Chinese Academy of Sciences, Beijing 100049, China

**Keywords:** coastal saline soil, shallow groundwater depth, alfalfa cultivars, forage yield, nutritional value

## Abstract

Freshwater shortage and soil salinization are the major constraints for alfalfa (*Medicago sativa* L.) growth in coastal salt–alkali soil of North China. In this study, we analyzed the effects of shallow groundwater tables and alfalfa cultivars on forage yield and nutritional value. A field simulation experiment was conducted during the growing season of 2019–2021 with three groundwater depths (80, 100, and 120 cm) and five alfalfa cultivars (Magnum 551, Phabulous, Zhongmu No. 1, Zhongmu No. 3, and WL525HQ) under subsurface pipe systems. Alfalfa forage was harvested six times in total during the growing season. Results revealed significant variation among alfalfa cultivars for forage yield at each shallow groundwater depth. The greatest forage yield was recorded in cultivar Phabulous (32.2 and 35.9 t ha^−1^ in 2020 and 2021) when planted at 100 cm shallow groundwater depth. Forage yield during the first harvest was 24.6–25.7%, exhibiting the highest ratio of the total annual yield. The effects of shallow groundwater depth, cultivar, and their interaction were significant (*p* < 0.01) on the turn-green ratio of alfalfa. Cultivar Zhongmu No. 1 had the highest turn-green ratio at the 100 cm groundwater depth, while cultivar WL525HQ showed the lowest turn-green ratio at each groundwater depth. Moreover, crude protein (CP), neutral detergent fiber (NDF), and acid detergent fiber (ADF) content were also significantly affected by shallow groundwater depth, cultivars, and their interaction at different harvests. Cultivars Magnum551, Zhongmu No. 1, Zhongmu No. 3, and Phabulous furnished the highest CP, while cultivar WL525HQ performed the poorest in terms of CP in this study. These results propose that planting the cultivar Phabulous at a groundwater depth of 100 cm could be a suitable agronomic practice for alfalfa forage production in the coastal salt–alkali area of North China.

## 1. Introduction

Soil salinization and alkalization have become more serious in numerous coastal regions throughout the world [1]. Saline–alkali soil is not only unfavorable for agricultural productivity, but is also unfavorable for water storage and soil nutrient availability, inevitably leading to drought [2]. Both drought and salt–alkali stress adversely influence crop growth via restricted water uptake and the presence of excessive salt [3,4]. Coastal saline–alkali land is an important cultivable land resource in China [5]. However, few plants can grow in this region due to the high soil salinity and freshwater scarcity [6,7]. Salinity stress affects crop water transpiration and water retention capacity [8]. Agricultural mismanagement and overexploitation of water resources in some arid or semiarid climates were important reasons for soil salinization [9]. The underlying saline groundwater in this area provides abundant water resources for agricultural production [10] and has a large potential for exploitation and use. At present, the use of shallow groundwater has become an important measure to relieve agricultural freshwater scarcity [11]. Crop water use from shallow groundwater will be a necessary practice to improve crop yields [12]. Thus, agricultural water demand will have to rely on shallow saline water to meet the crop water requirements in the future.

Shallow groundwater depth is also an important factor affecting alfalfa growth in the coastal saline soils of North China. Previous studies showed that plant water use is affected by plant species, water table depth, climate, and soil type [13,14]. The saline groundwater may affect plant root development. When surface water is insufficient, groundwater levels are to be maintained within a proper range [15,16]. Shallow groundwater can meet the seasonal crop water requirements. Many studies in literature report groundwater contribution for safflower (*Carthamus tinctorius* L.) varying between 52.5% and 54.9% [17,18]. Similarly, shallow groundwater contributes up to 50% of the daily crop water use for alfalfa [12]. The groundwater contribution for safflower was 72%, 70%, and 47% at groundwater table depths of 0.6, 0.8, and 1.1 cm, respectively [19]. Hence, more studies are needed to examine the effect of shallow groundwater table depth on the production performance and nutritional value of plants.

Alfalfa (*Medicago sativa* L.) is one of the most popular forage crops in the world because of its high content of crude protein and highly digestible fiber content [20,21,22,23], and it can be harvested many times during its growing season [24]. Moreover, cows absorb a significantly higher amount of potassium from alfalfa (89.0%) than other cereal plants (78.5%) [25]. Differences in forage yield and nutritional value of cultivars have been inconsistent and are greatly influenced by water conditions [26]. Alfalfa rooting depth of about 2–5 m has been reported in literature [27], and alfalfa planting could enhance the shallow groundwater use, reduce evapotranspiration ratio, and inhibit soil salinity [28,29]; thus, absorbing water and nutrient from deep soil layers, and increasing water use efficiency and for productivity. According to [27], groundwater depth has a significant effect on alfalfa root length. They report that the root depth was 0.42, 0.75, 1.13, and 1.30 m at the water table depths of 0.75, 1.05, 1.40, and 1.60 m, respectively. To address this challenge of water scarcity and soil salinization, it is necessary to develop alfalfa cultivars with high shallow groundwater use efficiency in the coastal region.

Based on the aforementioned contexts, it was hypothesized that shallow groundwater table depths and cultivars would significantly affect the biomass production and forage nutritional value. In order to more fully understand the variation of forage yield, nutritional value, and turn-green ratio in alfalfa, the objectives of this study were to the following: (1) compare the biomass production and forage nutritional value of alfalfa cultivars planted at different shallow groundwater depths; (2) reveal the effects of different shallow groundwater depths and cultivars on the turn-green ratio of alfalfa in the second year after planting.

## 2. Materials and Methods

### 2.1. Study Area and Site Description

**Figure 1:**
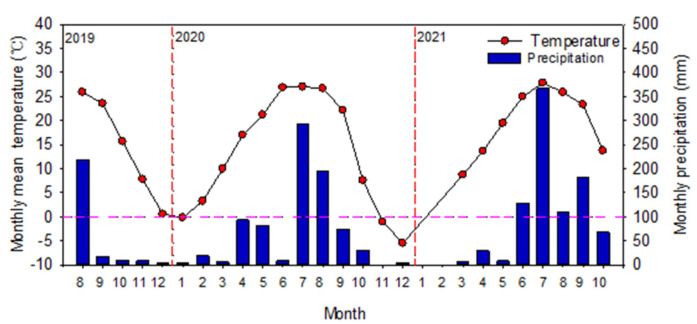
Dynamics of monthly mean air temperature and monthly precipitation data for August 2019 through October 2021 at Hebei, China.

The simulation experiment in the field was conducted from August 2019 to October 2021 in coastal region at Nandagang Experimental Station (117°22′ E, 38°28′ N), Huanghua County of Hebei province in Northern China. It is about 10 km away from the coastline of Bohai Bay. The climate of this region is continental monsoon temperate with a rainy summer and dry spring, autumn, and winter. The annual mean temperature is 12 °C. The average annual rainfall in this area is approximately 568 mm, of which 65% falls in the months of July to August. The average annual potential evaporation is 1981 mm, while the average annual hours of sunshine are 2810 h. The annual frost-free period is approximately 194 days. The groundwater table range was from 0.3 to 1.2 m. The weather data was obtained from the station in the vicinity of the experimental site operated by the China Meteorological Administration (Figure 1). Soil type is Salic Fluvisol [30]. Soil salt content ranged from 0.2% to 0.6%.

### 2.2. Experimental Design

**Figure 2:**
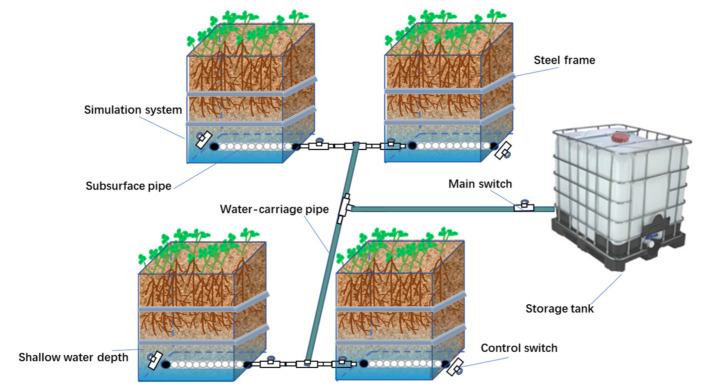
Simulation equipment of groundwater depth at Hebei, China.

Groundwater modeling system experiments were conducted to determine the effects of three shallow groundwater table depths and five alfalfa cultivars on the forage yield and quality. The tested alfalfa cultivars included Magnum 551, Phabulous, Zhongmu No. 1, Zhongmu No. 3, and WL525HQ. The source of tested cultivars is displayed in Table 1. The simulation experiment was arranged in a split-plot design with shallow groundwater table depths (D1, 80 cm; D2, 100 cm; D3, 120 cm) and alfalfa cultivars (C1, Magnum 551; C2, Zhongmu No. 1; C3, Zhongmu No. 3; C4, Phabulous; C5, WL525HQ) as the main plots and subplots, respectively. All treatments were replicated five times. The simulation equipment for the groundwater depth was made of 1.0 cm diameter polypropylene fiber composite plates. The rectangular simulation boxes were closed on the bottom and were connected to the plastic pipe via flexible polyvinyl chloride pipe. A plastic pipe and tank system were used to control the water table depth and to provide a volumetric measure of the amount of water required to maintain a relatively steady underground water level. The entire simulation box assembly was set on reinforced concrete slab, constructed of 1.5-m length, 1.5-m width, and 1.5-m height. The complete setup is shown in Figure 2.

The local air-dried saline soil was put into simulation boxes. The simulation boxes were packed to a bulk density of 1.3 g cm^−3^ with salty-alkaline soil collected from the 1.5 m depth of the soil profile near the experimental site. Water with low salt concentration was applied based on the change in the water volume of plastic tank. The simulation equipment used in this study determined the alfalfa water use from shallow groundwater depths.

For sowing, 2 g of seeds were planted on the furrows on 20 August 2019. The row spacing of alfalfa was kept 25 cm in the simulation boxes. Before sowing seeds, the base fertilizer was applied as manure (3.6 t ha^−^^1^), diammonium phosphate (800 kg ha^−1^; (N, 21.2% and P_2_O_5_, 53.8%), and potassium sulphate (180 kg ha^−1^; (K_2_O,54.1%). Weeds were pulled out manually all through the growing period. Alfalfa forage was harvested six times during the growing season at the height of 5-cm above the soil surface when 20% of the stems had flower buds. Stubble height for the last harvested alfalfa was 10 cm, which ensured alfalfa plant through winter safely. Six harvests were made on the following dates: 13 May, 10 June, 8 July, 4 August, 6 September, and 7 October 2020. In 2021, seven harvests were made on the following dates: 13 May, 6 June, 27 June, 20 July, 12 August, 11 September, and 17 October.

### 2.3. Sampling and Measurements

The number of turn-green plants in the simulation box was counted on 28 April 2020. Observed sprouting was considered to be turning green. All aboveground plants from simulation box in each experimental unit (3 depths × 5 cultivars × 5 replicates) were harvested and weighed separately to estimate the fresh forage yield. Afterwards, subsamples were weighed and then placed in forced air oven for 48 h at 65 °C and weighed again to determine the dry forage yield and yield proportion by harvest. Then, oven-dried samples were ground into a fine powder, passed through a 0.15 mm sieve, and analyzed for forage quality attributes, i.e., crude protein (CP), neutral detergent fiber (NDF), and acid detergent fiber (ADF), according to AOAC International Methodology [31]. The nitrogen content was determined by the Kjeldahl method [32] and CP content was calculated by the formula of N × 6.25 [33]. The NDF and ADF content were measured by the Van Soest method [34].

### 2.4. Statistical Analysis

Response variables include forage yield, CP, NDF, ADF, and turn-green ratio. The data for all treatments was presented on the basis of five replicated measurements. The analysis of variance (ANOVA) was performed using SAS 8.0 statistical software (SAS Institute, Cary, NC, USA). Mean comparisons between the treatments were tested by the Fisher’s least significant difference (LSD) test using 95% confidence interval [35]. Data were expressed as mean ± standard deviation (SD). All figures were created using Sigmaplot 12.5 (Systat Software, Inc., San Jose, CA, USA).

## 3. Results

### 3.1. Analysis of Variance

The effect of shallow groundwater table depth on CP, NDF, and ADF was significant (Table 2). Similarly, the effect of the alfalfa cultivar on total dry forage yield, CP, NDF, ADF, and turn-green ratio was also significant (*p* < 0.01). The groundwater table depth significantly affected the dry forage yield of the first, second, and sixth harvests, and the percent of dry forage yield of each harvest in the total yield (*p* < 0.01). Likewise, the effect of the cultivar on the dry forage yield of the first, third, and fifth harvests, and the percent of dry forage yield in all the harvests was significant (*p* < 0.05). The interaction between groundwater depth and cultivar was significant for all the studied parameters (*p* < 0.01).

### 3.2. Forage Yield Changes with Groundwater Depth and Cultivar

**Figure 3:**
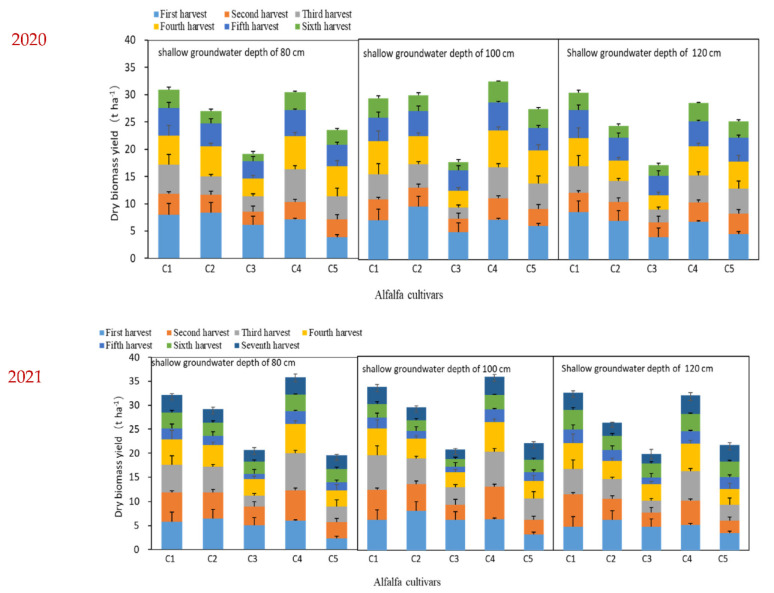
The dry biomass yield of each harvest of five alfalfa cultivars planted under three different groundwater depths in 2020 and 2021 at Hebei, China. Values are the means of five replicates. C1, Magnum551; C2, Zhongmu No. 1; C3, Zhongmu No. 3; C4, Phabulous; C5, WL525HQ.

Forage yield at a groundwater depth of 100 cm was higher compared with the groundwater depths of 80 cm and 120 cm in 2020 and 2021 (Figure 3). The sequence of the forage yield at different groundwater depths in 2020 was as follows: 100 cm > 120 cm > 80 cm. Alfalfa cultivar Magnum551 had the highest forage yield (30.9 t ha^−1^), which was approximately 60.9% higher than the value for cultivar Zhongmu No. 3 at groundwater depth of 80 cm. The forage yield of cultivar Phabulous was 81.9% higher than that of Zhongmu No. 3 at a groundwater depth of 100 cm. The highest and lowest forage yields were recorded for the cultivars Phabulous (32.2 t ha^−1^) and Zhongmu No. 3 (17.1 t ha^−1^) at a groundwater depth of 100 cm, respectively. In 2021, the average forage yield of five cultivars at a groundwater depth of 100 cm was 3.3% and 10.1% higher than that at groundwater depths of 80 and 120 cm, respectively. The average forage yield for Juneng551 and Phabulous was higher than that for the other three cultivars at all groundwater depths.

**Figure 4:**
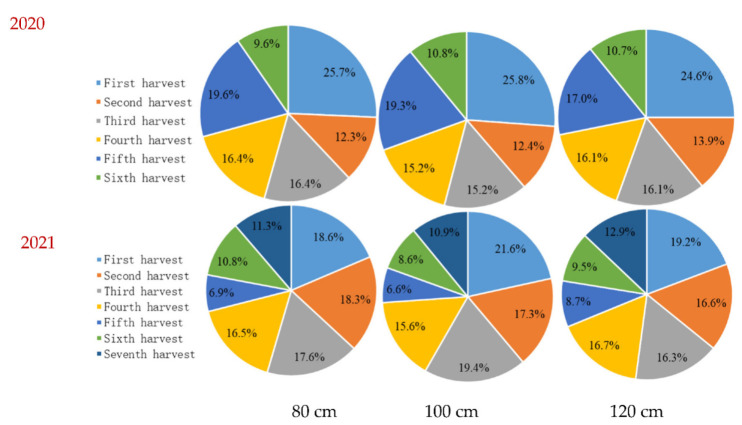
The percent of forage yield at each harvest in total annual yield under three different groundwater depths (viz., 80 cm, 100 cm, and 120 cm) in 2020 and 2021 at Hebei, China.

The highest forage yields of 25.7%, 25.8%, and 24.6% at groundwater depths of 80, 100, and 120 cm, respectively, were achieved from the first harvest in 2020 (Figure 4). The proportion of the sixth harvest in total forage yield was the lowest, being 16.1%, 15.0%, and 13.9% at the groundwater depths of 80, 100, and 120 cm, respectively. The sequence of forage yield proportion among the six harvests was as follows: first harvest > fifth harvest > third and fourth harvests > second harvest > sixth harvest. In 2021, the proportion of first, second, third, and fourth harvests in total forage yield had reached 71.0%, 73.9%, and 68.8% at the groundwater depths of 80, 100, and 120 cm, respectively.

### 3.3. Effects of Shallow Groundwater Depth and Cultivar on Turning Green of Alfalfa

**Figure 5:**
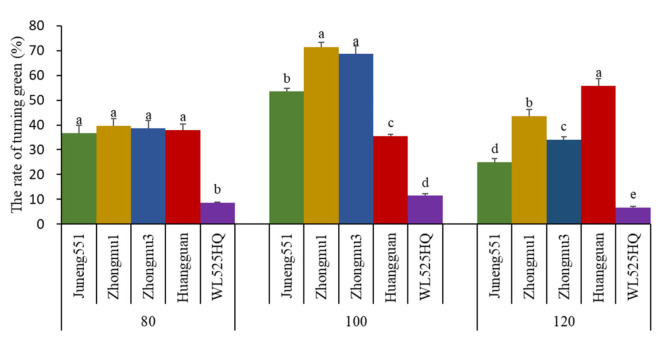
The effects of shallow groundwater table depth and cultivar on the returning green rate of alfalfa at Hebei, China. Values are the means of five replicates. 80: shallow groundwater depth of 80 cm; 100: shallow groundwater depth of 100 cm; 120: shallow groundwater depth of 120 cm. Data represent treatment means and standard deviation, *n* = 5. Different small letters indicate the significant differences among means at 0.05 significance level.

There was a significant difference in the rate of turning green among different alfalfa cultivars at the shallow groundwater table depths of 100 and 120 cm (*p* < 0.05) (Figure 5). According to the results, the average greening rate of all alfalfa cultivars with a groundwater depth of 100 cm was about 40.6% and 47.0% higher than that at the groundwater depths of 80 cm and 120 cm, respectively. Cultivar WL525HQ had the lowest greening rate (8.9%), which was approximately 3.8-, 4.8-, 4.3-, and 3.8-fold lower than the values of cultivars Magnum551, Zhongmu No. 1, Zhongmu No. 3, and Phabulous, prospectively.

It is of note that the turning green rate of cultivars Zhongmu No. 1 and Zhongmu No. 3 was better when compared to cultivars Magnum551, Phabulous, and WL525HQ at the shallow groundwater depths of 80 cm and 100 cm (Figure 5). Under the deeper groundwater depth (120 cm), the rate of turning green of the cultivars Zhongmu No. 1 and Phabulous had peak values of 43.5% and 55.7%, respectively, while those of cultivars Magnum551, Zhongmu No. 3, and cultivar Zhongmu No. 3 had minimum values of 25.0%, 34.1%, and 6.6%, respectively.

### 3.4. Effects of Groundwater Depth and Cultivar on Alfalfa Forage Quality

#### 3.4.1. Crude Protein

In the third harvest, Zhongmu No. 3 had the significantly highest CP content (2.03 g kg^−1^) at a groundwater depth of 80 cm (Table 3). At a groundwater depth of 100 cm, Zhongmu No. 3 (2.03 g kg^−1^) had the highest CP content, followed by cultivar Phabulous (2.00 g kg^−1^). During the fourth harvest, cultivar Phabulous had the highest CP content (2.07 g kg^−1^) at a groundwater depth of 80 cm. The CP content of cultivar Magnum551 was highest at a groundwater depth of 120 cm. During the fifth harvest, CP content of cultivar Magnum551 was significantly the highest at a groundwater depth of 100 cm.

**Figure 6:**
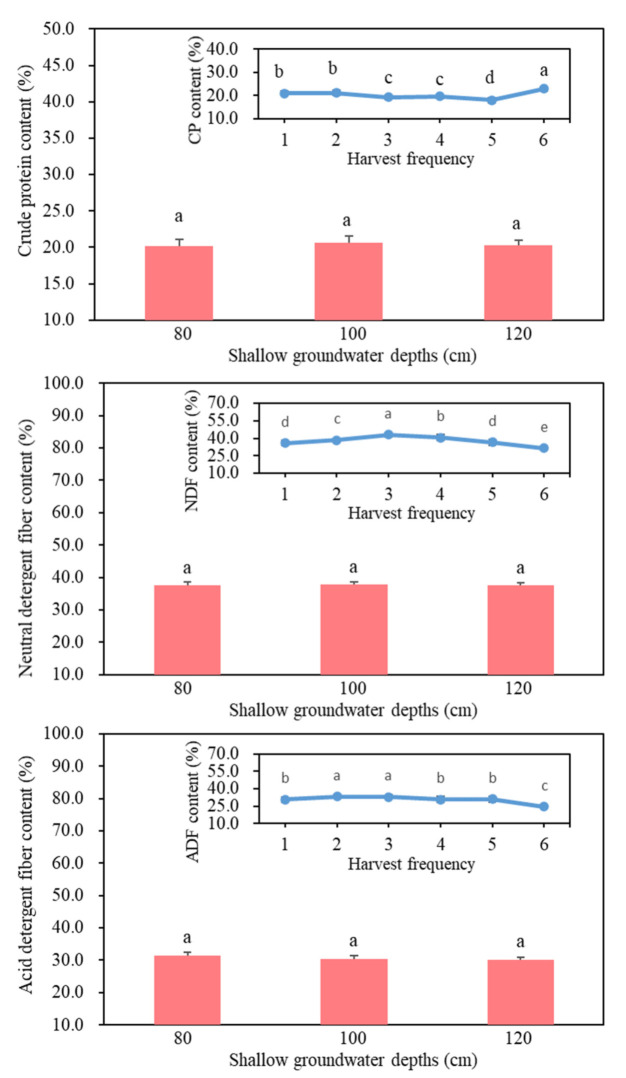
The effects of shallow groundwater depth and harvest frequency on the contents of crude protein (CP), neutral detergent fiber (NDF), and acid detergent fiber (ADF), and relative forage value (RFV) at Hebei, China. Data represent treatment means and standard deviation, *n* = 5; Different small letters indicate the significant differences among means at 0.05 significance level.

There was a significant difference in CP content among different harvest periods (*p* < 0.05) (Figure 6). The highest CP content of 2.28, 2.34, and 2.25 g kg^−1^ at groundwater depths of 80, 100, and 120 cm, respectively, was achieved from the sixth harvest. The lowest CP content of 1.85, 1.82, and 1.70 g kg^−1^ at groundwater depths of 80, 100, and 120 cm, respectively, was achieved from the fifth harvest.

#### 3.4.2. Neutral Detergent Fiber

At the first, second, and fifth harvests, no significant differences were observed in NDF among cultivars at all groundwater depths (Table 4). In the third harvest, the NDF content of cultivar WL525HQ was highest at a groundwater depth of 100 cm, while the NDF content of cultivar Magnum551 was significantly the lowest (Figure 6). During the fourth harvest, the NDF content of cultivar WL525HQ was highest at a groundwater depth of 100 cm, while the NDF content of cultivar Phabulous was lowest. Cultivar WL525HQ at a groundwater depth of 120 cm had the highest NDF content, which was 15.8% and 16.5% higher than that of Magnum551 and Phabulous, respectively. During the sixth harvest, the NDF content of cultivar WL525HQ was the significantly highest, while that of cultivar Phabulous was the lowest.

#### 3.4.3. Acid Detergent Fiber

During the first and second harvests at all groundwater depths, no significant differences were observed in the ADF content among alfalfa cultivars (Table 5). During the third harvest, at a groundwater depth of 100 cm, the ADF content of cultivar WL525HQ was significantly higher, while that of cultivar Phabulous was the lowest. During the fourth harvest, at a groundwater depth of 80 cm, WL525HQ had the significantly highest ADF, while the ADF content of cultivar Zhongmu No. 3 was the lowest (Figure 6). Cultivar WL525HQ was higher in ADF than rest of the cultivars at a groundwater depth of 120 cm. During the fifth harvest, cultivars WL525HQ and Zhongmu No. 3 furnished the highest (3.33 g kg^−1^) and lowest (2.96 g kg^−1^) ADF content, respectively, at a groundwater depth of 100 cm. The highest contents of ADF were noted in cultivar WL525HQ during the sixth harvest at all groundwater depths.

## 4. Discussion

### 4.1. Effects of Shallow Groundwater Depth, Cultivar, and Harvest Times on Alfalfa Dry Forage Yield

When alfalfa is planted in the saline soils of North China, the goal of a consistent supply of high-yielding and quality forage is limited by freshwater shortage and soil salinity. Groundwater is an important water source for alfalfa growth in shallow groundwater table areas. Shallow groundwater had a great influence on the distribution of water in different soil layers and alfalfa production, and the interaction between groundwater depth and alfalfa cultivars was significant (Table 1). The mean forage yield at 100 cm groundwater depth was noticeably higher than at groundwater depths of 80 and 120 cm (Figure 2), probably resulting from the greater use of shallow groundwater by alfalfa, which translated to lower leaching fractions [12,29]. Another reason could be that the roots of alfalfa are mostly distributed in the 0–100 cm deep soil layer [35]. The forage yield during the first harvest was the highest at the 100 cm groundwater depth, accounting for 25.8% and 21.6% of the annual total forage yield in 2020 and 2021, respectively (Figure 4). The high ratio of forage yield in this study region during the first harvest was attributed to the longer growth period than the subsequent harvests, which also reflects better water use for alfalfa grown in a dry period [36,37]. Furthermore, because of the low temperature and low shading, a large number of fresh leaves were formed, and they increased the leaf/stem ratio [38]. On the contrary, the low dry forage yield of alfalfa for the second harvest in 2020 was most probably due to the long-term dryness and high temperature before harvesting during the growing season, compared with 2021 (Figure 2). To maintain a higher alfalfa yield, our results suggest that Magnum551 and Phabulous could prove the best cultivars to be planted in this saline region of North China at a shallow groundwater depth of 100 cm (Figure 3).

### 4.2. Effects of Shallow Groundwater Depth, Cultivars, and Harvest Times on Alfalfa Nutritive Value

Many studies have reported that forage quality is increased by increasing the CP and RFV, while quality is decreased with increased NDF and ADF content [25,39]. In the present study, a high content of CP was observed in the first harvest. During subsequent harvests, a significant decrease in CP content from the second to fourth harvests was observed, but the highest CP content was noticed in the fifth and sixth harvests (Table 3). This rise in forage CP content is attributed to a weather-induced increase in the ratio of leaf to stem [33]. The nitrogen content of alfalfa stems is low, while the leaves are rich in nitrogen [26]. Thus, increasing the leaf area led to higher CP levels of forage.

In this study, the percentage of crude fiber (NDF and ADF) for cultivar WL525HQ was recorded as the highest, as reported by another researcher for the same cultivar [40]. For instance, reduction of 0.8% and 1.3% in NDF and ADF content was found in the cultivars WL525HQ and Zhongmu No. 2, respectively [40]. In our case, NDF and ADF first increased, and then decreased with increasing harvest frequency, which could also be attributed to an increase or decrease in the leaf/stem ratio [25]. The NDF content increased from 18.6 to 42.6% between the first and fifth harvests of alfalfa, respectively; this has also been reported by Robinson [41].

Alfalfa cultivars and harvest times appear to be the major factors influencing forage quality, indicating that the nutritional value of alfalfa increased slightly with the increasing leaf/stem ratio [40]. The high temperature and precipitation mainly increased plant height and growth rate [42]. The forage quality of dry forage also suggested that cultivar WL525HQ was lower in terms of quality performance than the other four tested cultivars. Forage quality differences may be attributed to the differences in weather conditions (i.e., temperature and precipitation) and soil type. Less precipitation is not favorable for alfalfa nutritional point of view in arid regions; meanwhile, shallow groundwater can provide optimum level of moisture for alfalfa growth when the precipitation is not sufficient [43]. Hence, water stress resulted in reduced forage quality in their study.

## 5. Conclusions

The present study evaluated the forage yield, returning green rate, and forage nutritional value of five alfalfa cultivars grown at three shallow groundwater depths in a coastal saline area. The forage yield at a groundwater depth of 100 cm was higher than that at groundwater depths of 80 and 120 cm. Cultivar Phabulous furnished the highest forage yield at a groundwater depth of 100 cm. The proportion of forage yield from the first to fourth harvests in the total annual yield was over 65%. The turn-green ratio of cultivars Zhongmu No. 1 and Zhongmu No. 3 was the highest at a groundwater depth of 100 cm. The forage nutritional value (CP, ADF, and NDF) was significantly affected by the shallow groundwater table depth, cultivar, and their interaction. Based on the outcomes of this study, it is concluded that planting cultivars Magnum551, Zhongmu No. 1, and Phabulous at a groundwater depth of 100 cm appears to be a desirable practice for optimum forage yield and quality and a returning green ratio when alfalfa is being cultivated in saline soil. Further studies involving the variation of groundwater depth and salt content are required to confirm the shallow groundwater extraction and utilization capacity of salt-stressed alfalfa under field conditions.

## Figures and Tables

**Table 1:** Alfalfa cultivars and their origin.

Code	Cultivar’s Name	Fall Dormoncy Level	Seed Source
1	Magnum551	5.0	Beijing Clover Seed Industry Co., Ltd.
2	Zhongmu No. 1	3.0	Institute of Animal Science, CAAS
3	Zhongmu No. 3	3.0	Institute of Animal Science, CAAS
4	Phabulous	4.0	Beijing Clover Seed Industry Co., Ltd.
5	WL525HQ	8.0	Beijing Rytway Ecotechnology Co., Ltd.

**Table 2:** Statistical results of groundwater table depth, alfalfa cultivar, and their interaction at Hebei, China.

Factor	Annual Total Yield	CP	NDF	ADF	Turn-Green Ratio
Shallow groundwater depth (A)	14.7 (0.9493)	29.3 (0.0036 **)	283.6 (<0.0001 ***)	93.9 (<0.0001 ***)	6.6 (0.9493)
Alfalfa cultivar (B)	14.7 (<0.0001 ***)	141.6 (<0.0001 ***)	402.8 (<0.0001 ***)	42.2 (<0.0001 ***)	241.7 (<0.0001 ***)
A * B	7.6 (<0.0001 ***)	90.3 (<0.0001 ***)	119.3 (<0.0001 ***)	358.4 (<0.0001 ***)	605.7 (<0.0001 ***)
		**Forage Yield of Each Harvest**		**Percent of Forage Yield in Each Harvest**	
Shallow groundwater depth (A)	First harvest	183.1 (<0.0001 ***)		6.6 (0.9493)	
	Second harvest	64.5 (<0.0001 ***)		6.6 (0.9493)	
	Third harvest	6.6 (0.9215)		6.6 (0.9493)	
	Fourth harvest	18.3 (0.1083)		6.6 (0.9493)	
	Fifth harvest	17.4 (0.0668)		6.6 (0.9493)	
	Sixth harvest	169.5 (<0.0001 ***)		77.2 (<0.0001 ***)	
Alfalfa cultivar (B)	First harvest	179.0 (<0.0001 ***)		277.2 (<0.0001 ***)	
	Second harvest	5.4 (0.7118)		157.3 (<0.0001 ***)	
	Third harvest	461.5 (<0.0001 ***)		167.9 (<0.0001 ***)	
	Fourth harvest	1.1 (1.0000)		167.9 (<0.0001 ***)	
	Fifth harvest	40.2 (<0.0001 ***)		232.4 (<0.0001 ***)	
	Sixth harvest	27.4 (0.0023 **)		12.7 (<0.0001 ***)	
A * B	First harvest	420.1 (<0.0001 ***)		46.5 (<0.0001 ***)	
	Second harvest	45.7 (<0.0001 ***)		111.6 (<0.0001 ***)	
	Third harvest	531.8 (<0.0001 ***)		158.7 (<0.0001 ***)	
	Fourth harvest	0.0 (1.0000)		158.7 (<0.0001 ***)	
	Fifth harvest	447.5 (<0.0001 ***)		584.1 (<0.0001 ***)	
	Sixth harvest	221.8 (<0.0001 ***)		12.0 (1.0000)	

*, ** and ***: Significant correlation at the 5, 1, and 0.1% levels of probability, respectively. CP: crude protein, NDF: Neutral detergent fiber, ADF: acid detergent fiber.

**Table 3:** Crude protein content (g kg^−1^) of the dry forage as affected by shallow groundwater depth and cultivar at each harvest in 2020 at Hebei, China.

Treatment		First Harvest	Second Harvest	Third Harvest	Fourth Harvest	Fifth Harvest	Sixth Harvest	Average
Depths (cm)	Cultivars							
80	Magnum551	2.07 ± 0.24 a	2.08 ± 0.10 a	1.92 ± 0.15 ab	1.94 ± 0.10 a	1.92 ± 0.11 a	2.20 ± 0.07 a	2.02
	Zhongmu No.1	2.03 ± 0.40 a	2.06 ± 0.17 a	1.96 ± 0.07 ab	1.95 ± 0.07 a	1.82 ± 0.06 ab	2.36 ± 0.06 a	2.03
	Zhongmu No.3	2.00 ± 0.18 a	2.11 ± 0.33 a	2.03 ± 0.07 a	1.91 ± 0.09 a	1.89 ± 0.11 ab	2.26 ± 0.15 a	2.03
	Phabulous	2.12 ± 0.16 a	2.09 ± 0.11 a	1.92 ± 0.09 ab	19.6 ± 0.8 a	1.86 ± 0.11 ab	2.32 ± 0.10 a	2.04
	WL525HQ	2.02 ± 0.22 a	1.95 ± 0.16 a	1.84 ± 0.09 b	1.85 ± 0.16 a	1.74 ± 0.14 b	2.28 ± 0.32 a	1.95
	Average	2.05 B	2.06 B	1.93 C	1.92 CD	1.85 D	2.28 A	
100	Magnum551	2.02 ± 0.21 a	2.15 ± 0.9 a	1.98 ± 0.10 ab	1.99 ± 0.05 ab	1.88 ± 0.06 a	2.31 ± 0.11 a	2.06
	Zhongmu No.1	2.16 ± 0.14 a	2.09 ± 0.17 a	1.99 ± 0.10 ab	1.94 ± 0.08 ab	1.81 ± 0.16 a	2.42 ± 0.07 a	2.07
	Zhongmu No.3	2.07 ± 0.16 a	2.27 ± 0.17 a	2.00 ± 0.17 a	2.06 ± 0.07 a	1.87 ± 0.13 a	2.38 ± 0.16 a	2.11
	Phabulous	2.17 ± 0.18 a	2.07 ± 0.10 a	2.03 ± 0.22 a	2.07 ± 0.14 a	1.91 ± 0.11 a	2.32 ± 0.17 a	2.10
	WL525HQ	2.00 ± 0.10 a	2.03 ± 0.12 a	1.80 ± 0.02 b	1.85 ± 0.12 b	1.82 ± 0.16 a	2.28 ± 0.10 a	1.96
	Average	2.08 B	2.12 B	1.96 C	1.98 C	1.86 D	2.34 A	
120	Magnum551	2.21 ± 0.17 a	2.20 ± 0.14 a	1.88 ± 0.18 a	2.06 ± 0.09 a	1.79 ± 0.13 a	2.28 ± 0.04 a	2.07
	Zhongmu No.1	1.96 ± 0.13 a	2.25 ± 0.33 a	1.94 ± 0.10 a	1.96 ± 0.14 ab	1.73 ± 0.12 a	2.24 ± 0.06 ab	2.01
	Zhongmu No.3	2.25 ± 0.14 a	2.31 ± 0.42 a	1.97 ± 0.07 a	2.03 ± 0.17 ab	1.70 ± 0.10 a	2.32 ± 0.08 a	2.10
	Phabulous	2.24 ± 0.15 a	2.09 ± 0.19 a	1.87 ± 0.10 a	2.01 ± 0.13 ab	1.77 ± 0.16 a	2.22 ± 0.05 ab	2.03
	WL525HQ	2.17 ± 0.33 a	2.02 ± 0.19 a	1.84 ± 0.13 a	1.86 ± 0.12 b	1.53 ± 0.42 a	2.17 ± 0.10 b	1.93
	Average	2.17 A	2.17 A	1.91 B	1.98 B	1.70 C	2.25 A	

Values are the means of five replicates. For each harvest, different small letters in a column denote significant differences among means at *p* < 0.05. For average, different big letters in a line denote significant differences among means at *p* < 0.05.

**Table 4:** Neutral detergent fiber content (g kg^−1^) of the dry forage as affected by shallow groundwater depth and cultivar at each harvest in 2020 at Hebei, China.

Treatment		First Harvest	Second Harvest	Third Harvest	Fourth Harvest	Fifth Harvest	Sixth Harvest	Average
Depths (cm)	Cultivars							
80	Magnum551	3.74 ± 0.81 a	3.78 ± 0.16 a	4.34 ± 0.30 a	4.29 ± 0.28 a	3.16 ± 0.42 a	3.12 ± 0.23 a	3.74
	Zhongmu No. 1	3.86 ± 0.56 a	3.81 ± 0.31 a	4.18 ± 0.42 a	4.30 ± 0.30 a	3.41 ± 0.55 a	3.00 ± 0.58 a	3.76
	Zhongmu No. 3	3.92 ± 0.31 a	3.84 ± 0.32 a	4.17 ± 0.46 a	3.89 ± 0.79 a	3.38 ± 0.32 a	3.11 ± 0.62 a	3.72
	Phabulous	3.44 ± 0.31 a	3.79 ± 0.28 a	4.19 ± 0.27 a	4.28 ± 0.33 a	3.32 ± 0.47 a	3.12 ± 0.17 a	3.69
	WL525 HQ	3.39 ± 0.36 a	4.13 ± 0.29 a	4.41 ± 0.27 a	4.35 ± 0.44 a	3.71 ± 0.38 a	3.16 ± 0.76 a	3.86
100	Magnum551	3.58 ± 0.27 a	3.78 ± 0.17 a	3.90 ± 0.55 b	3.99 ± 0.13 b	3.75 ± 0.27 a	3.05 ± 0.22 ab	3.68
	Zhongmu No. 1	3.43 ± 0.44 a	3.80 ± 0.43 a	4.27 ± 0.24 ab	4.15 ± 0.13 ab	3.84 ± 0.27 a	3.11 ± 0.49 ab	3.77
	Zhongmu No. 3	3.78 ± 0.31 a	3.68 ± 0.38 a	4.39 ± 0.39 ab	3.97 ± 0.27 b	3.70 ± 0.23 a	2.94 ± 0.22 ab	3.74
	Phabulous	3.52 ± 0.22 a	3.78 ± 0.19 a	3.99 ± 0.41 ab	3.84 ± 0.21 b	3.89 ± 0.13 a	2.86 ± 0.42 b	3.65
	WL525 HQ	3.64 ± 0.12 a	4.07 ± 0.35 a	4.55 ± 0.32 a	4.38 ± 0.33 a	4.05 ± 0.34 a	3.39 ± 0.21 a	4.01
120	Magnum551	3.26 ± 0.18 a	3.67 ± 0.21 a	4.38 ± 0.16 a	3.66 ± 0.45 b	3.59 ± 0.21 a	3.24 ± 0.42 a	3.63
	Zhongmu No. 1	3.89 ± 0.28 a	3.97 ± 0.31 a	4.34 ± 0.46 a	3.82 ± 0.28 ab	4.00 ± 0.33 a	3.23 ± 0.55 a	3.88
	Zhongmu No. 3	3.51 ± 0.44 a	3.63 ± 0.68 a	4.41 ± 0.22 a	3.84 ± 0.31 ab	3.65 ± 0.39 a	3.23 ± 0.31 a	3.71
	Phabulous	3.38 ± 0.27 a	3.67 ± 0.27 a	4.50 ± 0.31 a	3.64 ± 0.15 b	3.44 ± 0.52 a	3.20 ± 0.18 a	3.64
	WL525 HQ	3.33 ± 0.50 a	3.96 ± 0.59 a	4.51 ± 0.42 a	4.24 ± 0.36 a	3.79 ± 0.62 a	3.44 ± 0.18 a	3.88

Values are the means of five replicates. For each harvest, different small letters in a column denote significant differences among alfalfa cultivars of the same groundwater depth at *p* < 0.05.

**Table 5:** Acid detergent fiber content (g kg^−1^) of the dry forage as affected by shallow groundwater depth and cultivar at each harvest in 2020 at Hebei, China.

Treatment		First Harvest	Second Harvest	Third Harvest	Fourth Harvest	Fifth Harvest	Sixth Harvest	Average
Depths (cm)	Cultivars							
80	Magnum551	3.08 ± 0.35 a	3.32 ± 0.11 a	3.20 ± 0.54 a	3.24 ± 0.18 ab	3.37 ± 0.33 a	2.47 ± 0.44 b	3.11
	Zhongmu No. 1	3.20 ± 0.33 a	3.21 ± 0.20 a	3.19 ± 0.15 a	3.09 ± 0.29 ab	3.39 ± 0.50 a	2.45 ± 0.15 ab	3.09
	Zhongmu No. 3	3.41 ± 0.39 a	3.34 ± 0.31 a	3.29 ± 0.44 a	2.99 ± 0.49 b	3.29 ± 0.32 a	2.49 ± 0.44 b	3.14
	Phabulous	2.96 ± 0.29 a	3.37 ± 0.27 a	3.21 ± 0.19 a	3.26 ± 0.29 ab	3.37 ± 0.25 a	2.52 ± 0.20 b	3.12
	WL525 HQ	2.93 ± 0.39 a	3.51 ± 0.14 a	3.51 ± 0.21 a	3.57 ± 0.46 a	3.45 ± 0.45 a	2.64 ± 0.55 a	3.27
100	Magnum551	3.14 ± 0.25 a	3.31 ± 0.17 a	3.24 ± 0.23 ab	3.01 ± 0.13 b	2.98 ± 0.22 b	2.45 ± 0.26 ab	3.02
	Zhongmu No. 1	3.01 ± 0.45 a	3.16 ± 0.04 a	3.16 ± 0.34 ab	3.06 ± 0.28 b	3.07 ± 0.13 ab	2.30 ± 0.15 b	2.96
	Zhongmu No. 3	3.32 ± 0.31 a	3.24 ± 0.35 a	3.42 ± 0.52 ab	2.80 ± 0.18 b	2.96 ± 0.20 b	2.23 ± 0.20 b	3.00
	Phabulous	3.06 ± 0.22 a	3.33 ± 0.22 a	3.02 ± 0.38 b	2.85 ± 0.28 b	2.98 ± 0.25 b	2.31 ± 0.33 b	2.93
	WL525 HQ	3.17 ± 0.11 a	3.50 ± 0.10 a	3.61 ± 0.19 a	3.45 ± 0.28 a	3.33 ± 0.30 a	2.71 ± 0.04 a	3.30
120	Magnum551	2.85 ± 0.20 a	3.24 ± 0.20 a	3.37 ± 0.33 a	2.83 ± 0.33 b	2.72 ± 0.30 a	2.41 ± 0.25 b	2.90
	Zhongmu No. 1	3.38 ± 0.29 a	3.41 ± 0.27 a	3.39 ± 0.50 a	2.97 ± 0.28 ab	3.13 ± 0.42 a	2.47 ± 0.12 b	3.13
	Zhongmu No. 3	3.06 ± 0.42 a	3.20 ± 0.66 a	3.29 ± 0.32 a	3.00 ± 0.28 ab	2.83 ± 0.29 a	2.52 ± 0.14 b	2.98
	Phabulous	2.96 ± 0.21 a	3.30 ± 0.25 a	3.37 ± 0.25 a	2.77 ± 0.16 b	2.79 ± 0.40 a	2.47 ± 0.16 b	2.95
	WL525 HQ	2.89 ± 0.49 a	3.47 ± 0.53 a	3.45 ± 0.45 a	3.33 ± 0.29 a	3.04 ± 0.14 a	2.81 ± 0.08 a	3.17

Values are the means of five replicates. For each harvest, different small letters in a column denote significant differences among alfalfa cultivars of the same groundwater depth at *p* < 0.05.

## Data Availability

The authors confirm that the data supporting the findings of this study are available within the article.
